# Combined Treatment With 2-(2-Benzofu-Ranyl)-2-Imidazoline and Recombinant Tissue Plasminogen Activator Protects Blood–Brain Barrier Integrity in a Rat Model of Embolic Middle Cerebral Artery Occlusion

**DOI:** 10.3389/fphar.2020.00801

**Published:** 2020-06-12

**Authors:** Linlei Zhang, Shasha Xu, Xiaoxiao Wu, Jiaou Chen, Xiaoling Guo, Yungang Cao, Zheng Zhang, Jueyue Yan, Jianhua Cheng, Zhao Han

**Affiliations:** ^1^Department of Neurology, The Second Affiliated Hospital and Yuying Children's Hospital of Wenzhou Medical University, Wenzhou, China; ^2^Department of General Intensive Care Unit, The Second Affiliated Hospital, College of Medicine, Zhejiang University, Hangzhou, China; ^3^Center of Scientific Research, the Second Affiliated Hospital and Yuying Children's Hospital of Wenzhou Medical University, Wenzhou, China; ^4^Department of Neurology, The First Affiliated Hospital of Wenzhou Medical University, Wenzhou, China

**Keywords:** 2-(-2-benzofuranyl)-2-imidazoline, blood–brain barrier, cerebral ischemia, recombinant tissue plasminogen activator, tight junction

## Abstract

Recombinant tissue plasminogen activator (rt-PA) is used to treat acute ischemic stroke but is only effective if administered within 4.5 h after stroke onset. Delayed rt-PA treatment causes blood-brain barrier (BBB) disruption and hemorrhagic transformation. The compound 2-(-2-benzofuranyl)-2-imidazoline (2-BFI), a newly discovered antagonist of high-affinity postsynaptic N-methyl-D-aspartate (NMDA) receptors, has been shown to have neuroprotective effects in ischemia. Here, we investigated whether combining 2-BFI and rt-PA can ameliorate BBB disruption and prolong the therapeutic window in a rat model of embolic middle cerebral artery occlusion (eMCAO). Ischemia was induced in male Sprague Dawley rats by eMCAO, after which they were treated with 2-BFI (3 mg/kg) at 0.5 h in combination with rt-PA (10 mg/kg) at 6 or 8 h. Control rats were treated with saline or 2-BFI or rt-PA. Combined therapy with 2-BFI and rt-PA (6 h) reduced the infarct volume, denatured cell index, BBB permeability, and brain edema. This was associated with increased expression of aquaporin 4 (AQP4) and tight junction proteins (occludin and ZO-1) and downregulation of intercellular adhesion molecule 1 (ICAM-1) and matrix metalloproteinases 2 and 9 (MMP2 and MMP9). We conclude that 2-BFI protects the BBB from damage caused by delayed rt-PA treatment in ischemia. 2-BFI may therefore extend the therapeutic window up to 6 h after stroke onset in rats and may be a promising therapeutic strategy for humans. However, mechanisms to explain the effects oberved in the present study are not yet elucidated.

## Introduction

Ischemic stroke seriously reduces quality of life and is a major cause of death and long-term disability worldwide (Hou and MacManus, 2002; Lo et al., 2003; Hou et al., 2008; Moskowitz et al., 2010). The most effective treatment for acute ischemic stroke is thrombolysis with intravenous recombinant tissue plasminogen activator (rt-PA); However, at most medical centers, rt-PA is effective only when used no later than 4.5 h after stroke onset (Hacke et al., 2008; Blinzler et al., 2011). Because rt-PA may have pleiotropic actions in the brain, including clot lysis, the vascular endothelium activation, intracellular Ca2+ accumulation (Oka et al., 2009). Delayed rt-PA is associated with severe complications such as disruption of the blood-brain barrier (BBB), increased risk of hemorrhagic transformation and brain edema. Studies show that this may be due to destruction of extracellular matrix components and increased BBB permeability, which can lead to secondary injury during reperfusion (Lo, 2008). Because of this, rt-PA treatment is administered to fewer than 5% of stroke victims (Feigin et al., 2014). There is an urgent need to develop neuroprotective agents that can prolong the therapeutic window of rt-PA so that more patients can be treated.

The compound 2-(2-benzofu-ranyl)-2-imidazoline (2-BFI) is a newly discovered high-affinity imidazoline I_2_ receptor (I_2_R) agonist and a non-competitive ligand of N-methyl-d-aspartate receptors (NMDARs). Over-stimulation of NMDA receptors with glutamate results in calcium overload leading to the activation of potentially neurotoxic mechanisms, such as calpain, which breaks down intracellular structural proteins (Jiang et al., 2010). 2-BFI attenuates excitotoxicity-mediated neuronal death and cerebral ischemia *in vivo* by inhibiting excessive calcium influx (Milhaud et al., 2000; Milhaud et al., 2002; Dong et al., 2008). We previously reported that 2-BFI can reduce endothelial cell damage, preserve cerebral vascular function, and mitigate BBB damage after acute ischemic stroke *in vitro* and *in vivo* (Tian et al., 2018; Zhang et al., 2018b). Studies show that this neuroprotective effect may be due to inhibition of apoptosis in endothelial and neuronal cells (Han et al., 2012). 2-BFI reduced expression of neuronal injury markers such as apoptosis-inducing factor and inflammatory cytokines in a rat model of autoimmune encephalomyelitis (Zhu et al., 2015). Recent studies have shown that 2-BFI also provided Neuroprotection against inflammation and necroptosis in traumatic brain injury (Ni et al., 2019).

We previously showed that 2-BFI transiently, reversibly, and noncompetitively blocks NMDARs, inhibiting glutamate-mediated excessive calcium influx, similar to the noncompetitive NMDAR antagonist memantine (Han et al., 2013). It has a relatively fast off-rate and transiently blocking hyperactive NMDARs reduces their ability to perturb normal physiological function (Johnson and Kotermanski, 2006). These results suggest that 2-BFI may preserve BBB integrity as effective therapeutics in reducing excitotoxicity-evoked ischemic brain injury. Therefore, it is believed that 2-BFI promises to be potential candidates.

In this study, we used a rat model of ischemia with embolic middle cerebral artery occlusion (eMCAO) to investigate whether combining 2-BFI and rt-PA preserves BBB integrity and prolongs the therapeutic window compared to rt-PA alone.

## Materials and Methods

### Experimental Animals

All animal experiments were approved by the Institutional Animal Care and Use Committee of Wenzhou Medical University and conducted following US National Institutes of Health (NIH) guidelines. Adult male Sprague Dawley rats (SLAC Laboratory Animals, Shanghai, China) were fed ad libitum and housed under a 12-h light-dark cycle. Males were chosen because estrogen in female rats can influence the cerebral infarction area. All animals were healthy and weighed between 250-280 g at the start of the experiment.

### eMCAO

Ischemia was induced by eMCAO as previously described (Overgaard et al., 1992; Zhang et al., 1997). This model closely mimics human ischemic stroke and is suitable for preclinical investigation of thrombolytic therapy. To prepare the blood clot, jugular venous sinus blood from a donor rat was drawn into a 20-cm piece of PE-50 tubing, held for 2 h at room temperature and then stored for 22 h at 4°C. A 5-cm section of the tube containing clotted blood was cut, and a single clot was then transferred into saline-filled PE-50 tubing with an outer diameter of 0.30-0.34 mm. Rats were anesthetized with 3% isoflurane and maintained with 1% isoflurane using an animal anesthesia apparatus (RWD Life Science, Shenzhen, China). The common carotid artery, external carotid artery, and internal carotid artery were separated, and the PE-50 tubing was advanced from the external carotid artery into the lumen of the internal carotid artery, until the tip of the catheter reached the origin of the middle cerebral artery. Using a PE-50 catheter, we placed a single homologous blood clot at the origin of the middle cerebral artery. A scalp incision was made, and a burr hole (1.5-mm diameter) was made in the left temporal bone (located at 2 mm posterior and 5 mm lateral to bregma) using a 0.7 mm spherical stainless steel burr, thereby exposing the dura. The probe was then immobilized at 0.5 mm above the dural surface. Regional cerebral blood flow (rCBF) was continuously monitored with a Doppler blood flow detector (Periflux System 5000; Perimed AB, Stockholm, Sweden) from 0 to 60 min after embolization and at 60 min after rt-PA thrombolytic therapy (Jin et al., 2014) to confirm successful eMCAO, which was defined as >70% reduction in rCBF. Rats in the sham group underwent the same operation but without injection of a blood clot.

### Thrombolytic Therapy and 2-BFI Infusion

152 ischemic rats were randomly separated into treatment and control groups. Rats in the treatment group were further split into 5 groups and treated with rt-PA alone at 3 h (early) or 6 h (late), 2-BFI alone at 0.5 h, or 2-BFI at 0.5 h combined with rt-PA at 6 or 8 h. The number of animals in each group is listed in **Table 1**. All drugs were given by intravenous infusion through the jugular venous sinus. Based on our previous study, 2-BFI (Tocris Bioscience, Bristol, UK) was infused at a dose of 3 mg/kg (Han et al., 2010). Recombinant human rt-PA (Genentech, San Francisco, CA) was infused intravenously at a dose of 10 mg/kg (10% within 1 min, then 90% within 29 min) using a syringe infusion pump (BYZ-810; Tongsheng Yida Medical Technology, Beijing, China) (Zhu et al., 2010). This rt-PA dose is routinely used for investigating the effect of fibrinolysis in small rodents (Niessen et al., 2002). Controls were infused with 0.9% saline at 0.5 h. Rectal temperature was maintained at 37°C using a controlled heating pad. Thrombolysis was considered successful if rCBF returned to >70% of baseline within 60 min of rt-PA therapy.

**Table 1:** Experimental groups.

Group	Total (n)	Excluded (n)	Died (n)	Included (n)	Mortality (%)
Sham	24	0	0	24	0
eMCAO	34	2	8	24	24
eMCAO+rt-PA (3 h)	9	0	1	8	11
eMCAO+2-BFI (0.5 h)	29	2	3	24	10
eMCAO+rt-PA (6 h)	48	6	18	24	38
eMCAO+2-BFI (0.5 h)+rt-PA (6 h)	32	5	3	24	9
eMCAO+2-BFI (0.5 h)+rt-PA (8 h)	37	7	6	24	16

### Measurement of Neurological Dysfunction

Neurological function was assessed using the modified Neurological Severity Score (mNSS) at 30 h after surgery. Assessors were blinded to treatment group (Seyfried et al., 2004). The mNSS score ranges from 0 to 17, where higher scores indicate more serious neurological deficits.

### Measurement of Infarct Volume

**Figure 1:**
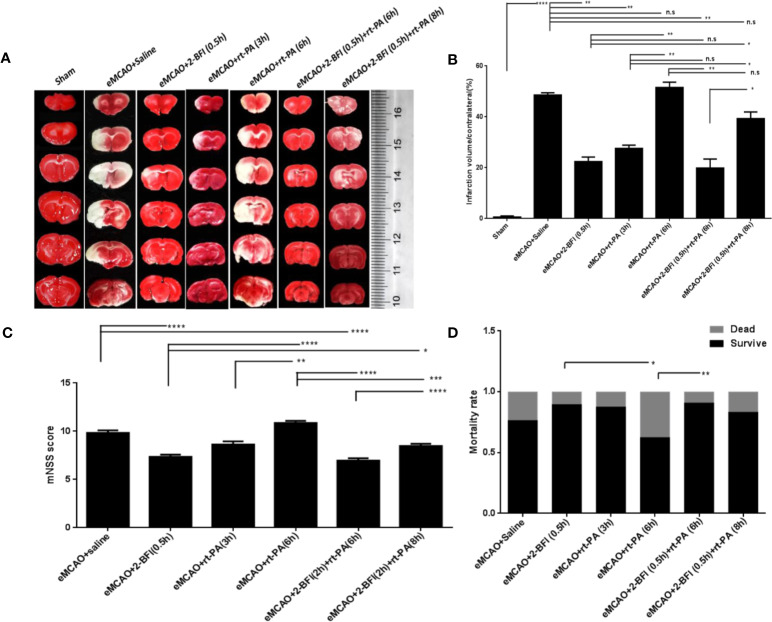
Combining 2-BFI + rt-PA provides better protection against neuronal damage than rt-PA alone. **(A)** TTC-stained brain sections of eMCAO rats treated with 2-BFI, rt-PA or 2-BFI + rt-PA. Infarct areas appear white. **(B)** Quantification of infarct volumes (n = 6) as follow: percentage of corrected infarct areas = (volume of total contralesional hemisphere-volume of intact ipsilesional hemisphere) *100% / contralateral hemispheric volume (Jiang et al., 2005; Hou et al., 2009). **(C)** Neurological function in eMCAO rats treated with 2-BFI, rt-PA or 2-BFI + rt-PA (n = 8-24 per group). **(D)** Mortality rates in eMCAO rats treated with 2-BFI, rt-PA or 2-BFI + rt-PA (n = 9-37 per group). Results are presented as mean ± SEM. ^*^p < 0.05, ^**^p < 0.01, ^***^p < 0.001, ^****^p < 0.0001; n.s., not significant.

Rats were euthanized at 30 h after surgery by intraperitoneal injection with 1% pentobarbital sodium (40mg/kg). The brains were removed, cut into 2-mm coronal sections using a rat brain mold (RWD Life Science, Shenzhen, China), stained with 2% 2,3,5-triphenyltetrazolium chloride (TTC, Sigma, MO, USA) at 37°C for 20 min, and fixed with 4% paraformaldehyde. The infarct area was measured in the neocortex and striatum of each section using Image-Pro Plus 6.0 software (Media Cybernetics, Rockville, MD, USA). The researcher measuring infarct area was blinded to treatment group. To minimize edema-induced error, infarct volumes were measured in the ischemic region as follows: percentage of corrected infarct areas = (volume of total contralesional hemisphere-volume of intact ipsilesional hemisphere) *100%/contralateral hemispheric volume (Jiang et al., 2005; Hou et al., 2009) (**Figure 1B**).

### Hematoxylin and Eosin Staining

Rats were anesthetized using 1% pentobarbital sodium, and the heart was exposed. An incision was made in the right ventricle and a syringe was placed inside. The animal was then perfused with normal saline followed by 50 mL of 4% paraformaldehyde in phosphate-buffered saline (PBS). The brain was removed after perfusion, immersed in 4% paraformaldehyde for 24 h and fixed and sectioned into 5-µm slices. Sections were stained with hematoxylin and eosin (H&E) as previously described (Zhang et al., 2018b) and observed under a Bx51 light microscope (Olympus, Tokyo, Japan) at ×400 magnification. Nerve damage was assessed using the denatured cell index, which was calculated as the number of degenerated cells/total number of cells × 100%.

### Brain Water Content

Rats were anesthetized with 1% pentobarbital sodium and the brains were bisected into right and left hemispheres. The left hemisphere was weighed immediately on an electronic balance to obtain wet weight and then dried in an oven at 65°C for 72 h. The brains were then re-weighed to obtain dry weight. The brain water content was calculated as: (wet weight – dry weight)/wet weight × 100% (Agrawal et al., 1968; Li et al., 2009).

### Evans Blue Extravasation

Rats were anesthetized with 1% pentobarbital sodium at 28 h after eMCAO. BBB integrity was measured using Evans blue dye staining as previously described (Liu et al., 2013). Evans blue (2%, Sigma) was quantified using a UV-6100S spectrophotometer (Meipada Instrument Co, Shanghai, China).

### Western Blot

Proteins were extracted from the ischemic penumbra of rat brains by radioimmunoprecipitation assay buffer (Pierce Chemical Co, Rockford, IL, USA) as previously described (Guo et al., 2016). A 40 μg sample of protein was then boiled by heating to 100°C for 10 min, fractionated by electrophoresis on a 10% sodium dodecyl sulfate-polyacrylamide gel, and transferred to a 0.45-μm polyvinylidene fluoride membrane (Bio-Rad, Hercules, CA, USA). The membrane was blocked with 5% nonfat milk for 1.5 h, washed and incubated overnight at 4°C with primary antibodies against AQP-4, ICAM-1, MMP2, MMP9, Occludin (all diluted 1:1000; Abcam, UK), ZO-1 (1:1000; Santa Cruz Biotechnology), or GAPDH (1:5000; Bioworld Technology, MN, USA). The next day the membrane was washed again and incubated with horseradish peroxidase-conjugated secondary anti-rabbit or anti-mouse antibodies (1:3000; Bioworld Technology, MN, USA) for 1 h at room temperature. Bands were visualized by enhanced chemiluminescence (Pierce Chemical, Rockford, IL, USA) and quantified using ImageJ software (NIH, Bethesda, MD, USA).

### Quantitative Reverse Transcription PCR (qRT-PCR)

Total RNA was extracted from infarcted brain tissue using TRIzol (Invitrogen, Carlsbad, CA, USA) according to the manufacturer's protocol and treated with Baseline-Zero DNase (Epicentre, Madison, WI, USA) to remove contaminating genomic DNA. RNA (2 μg) was reverse-transcribed using the RevertAid First Strand cDNA Synthesis kit (Applied Biosystems, Foster City, CA, USA). Quantitative RT-PCR was performed using SYBR Green PCR Master Mix (Applied Biosystems) and primers against AQP-4, ICAM-1, MMP2, MMP9, and GAPDH (see **Table 2** for primer sequences). Cycling was performed in a LightCycler 480 Quantitative Real-Time PCR System (Applied Biosystems) using the following conditions: 2 min at 50°C and 10 min at 95°C, followed by 45 cycles of 15 s at 95°C and 1 min at 62°C. The fluorescence threshold value (Ct value) was determined using Applied Biosystems software, and mRNA expression levels were calculated relative to GAPDH using the ΔΔCt method.

**Table 2:** Primer sequences.

Gene name	Primer direction	Sequence (5 ‘ to 3 ‘)	PCR (bp)	Accession no.
*MMP2*	Forward	CAGTACCAGTGTCAGTATCAGCAT	90	NM_031054.2
	Reverse	CCAAGAACTTCCGACTATCCA		
*MMP9*	Forward	CAAGGATGGTCTACTGGCACACG	158	NM_031055.1
	Reverse	AGGTGAAGGGAAAGTGACATGGG		
*ICAM-1*	Forward	CTGGAGAGCACAAACAGCAGAGAT	161	NM_012967.1
	Reverse	ATGGGAGCTGAAAAGTTGTAGATTC		
*AQP4*	Forward	GTGGGTGTGGAAACAAAGAGCAT	104	NM_001270559.2
	Reverse	CTGGCAGAGCCGTGAGTGAA		
*GADPH*	Forward	TCTCTGCTCCTCCCTGTTC	87	NM_017008.4
	Reverse	ACACCGACCTTCACCATCT		

### Gelatin Zymography

Total protein was extracted from the ischemic penumbra of the ipsilateral hemisphere. Gelatin zymography was performed as previously described (Fujimura et al., 1999). The MMP2 and MMP9 bands were analyzed using Image J software.

### Statistical Analysis

All statistical analysis was performed using SPSS 17.0 (SPSS, Chicago, IL, USA). Data are reported as mean ± SEM. Differences among more than two groups were assessed using one-way analysis of variance (ANOVA), followed by the Scheffe test. Inter-group differences in mortality rate were assessed using the chi-squared test. A *p*-value of < 0.05 was considered statistically significant. Data were graphed using Image-Pro Plus 6.0 and Prism 6.0 (GraphPad Software, San Diego, CA, USA).

## Results

### Combining 2-BFI and rt-PA Provides Better Protection Against Neuronal Damage Than rt-PA Alone

Control rats treated with saline after eMCAO had large infarct areas in the ipsilateral hemisphere, which were not observed in sham-operated rats (**Figure 1A**). As expected, rats treated with rt-PA at 3 h after eMCAO had much smaller infarct volumes than controls treated with saline, while there was no difference in infarct volume between rats given delayed rt-PA at 6 h and controls (**Figure 1A**). In contrast, rats treated with 2-BFI alone or with 2-BFI + rt-PA (6 or 8 h) had smaller infarct volumes than controls. The infarct volume was much smaller for those treated with 2-BFI + rt-PA (6 h) than 2-BFI + rt-PA (8 h).

Assessment of neurological deficits after treatment showed no difference in mNSS scores between rats treated with rt-PA alone at 3 h or 6 h and saline-treated controls. However, rats treated with 2-BFI alone or 2-BFI + rt-PA (6 h) had lower mNSS scores than controls treated with saline (**Figure 1C**). There was no difference between rats treated with 2-BFI + rt-PA (8 h) and controls. The mortality rate of rats treated with 2-BFI + rt-PA (6 h) was also lower than that of rats treated with rt-PA (6 h) alone (**Figure 1D**). Collectively, these results show that combining 2-BFI with rt-PA (6 h) provides much better protection against infarction and neurological damage than rt-PA alone.

### Combining 2-BFI and rt-PA Promotes Cerebrovascular Integrity

**Figure 2:**
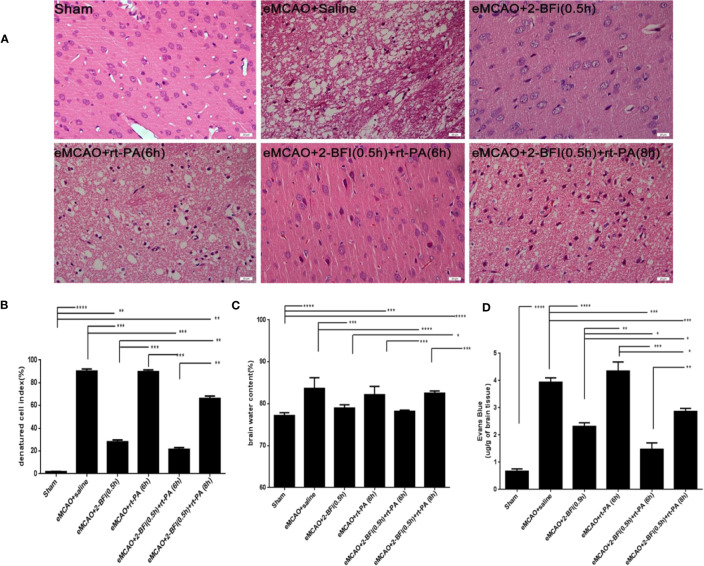
Combining 2-BFI and rt-PA reduces brain edema and Evans blue extravasation. **(A)** H&E-stained cortical tissue in eMCAO rats treated with 2-BFI, rt-PA or 2-BFI + rt-PA (scale bar = 20 μm). Quantitative analysis of **(B)** the denatured cell index (n = 4), **(C)** brain edema content in the ipsilateral hemisphere (n = 5), and **(D)** Evans blue extravasation (n = 4) in eMCAO rats treated with 2-BFI, rt-PA or 2-BFI + rt-PA. Results are shown as mean ± SEM. ^*^p < 0.05, ^**^p < 0.01, ^***^p < 0.001, ^****^p < 0.0001.

Cells in the cortical infarct region of saline-treated controls exhibited numerous characteristics of ischemic neuronal injury, including cell shrinkage, fragmented nuclear pyknosis, and increased extracellular space. Treatment with 2-BFI alone or 2-BFI+ rt-PA (6 h) ameliorated the cellular changes associated with ischemic neuronal injury and reduced the denatured cell index compared to saline-treated controls (**Figure 2A**). In contrast, there was no difference in denatured cell index between rats treated with rt-PA (6 h) alone and saline-treated controls (**Figures 2A, B**). Disruption of the BBB after thrombolytic treatment is predictive of brain edema in the eMCAO model (Ma et al., 2018). Brain water content was lower in rats treated with 2-BFI alone or 2-BFI + rt-PA (6 h) than in saline-treated controls, suggesting reduced edema. Brain water content was also lower in rats treated with 2-BFI + rt-PA (6 h) than in those treated with rt-PA alone (**Figure 2C**). There was no difference in brain water content between any of the groups in the hemisphere unaffected by stroke (data not shown). Next, we further assessed BBB permeability using Evans blue extravasation at 30 h after stroke (24 h after delayed rt-PA). Evans blue dye was present only in low levels in the brain parenchyma of sham animals, but dramatically increased in rats treated with saline or rt-PA alone (**Figure 2D**). Importantly, rats treated with 2-BFI + rt-PA at either 6 or 8 h had lower levels of Evans blue dye than saline-treated controls and rats treated with rt-PA alone, indicating that combining 2-BFI with rt-PA protects BBB integrity.

### BFI and rt-PA Increase Expression of Tight Junction Proteins

**Figure 3:**
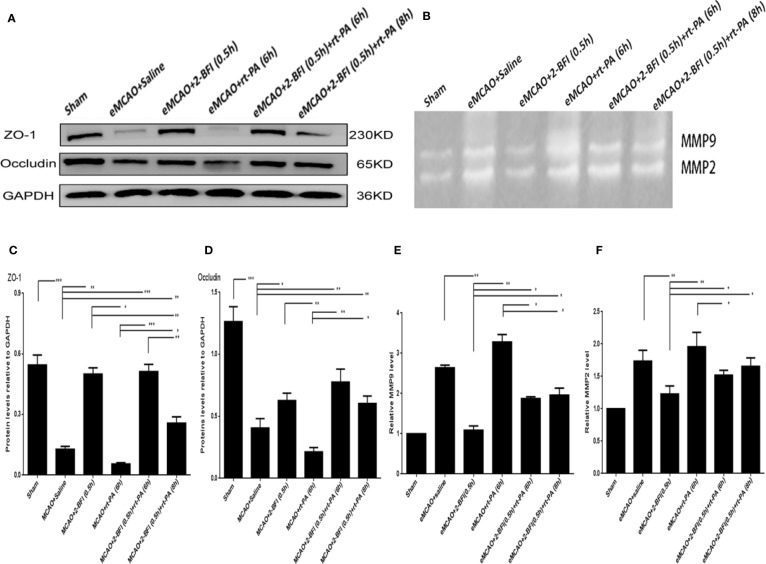
Combining 2-BFI and rt-PA rescued expression of tight junction proteins and expression of activity of MMP9/2 proteins in rats subjected to eMCAO. **(A)** Expression of Occludin and ZO-1 in eMCAO rats treated with 2-BFI, rt-PA or 2-BFI + rt-PA as assessed by western blot. **(B)** MMP9 and MMP2 activation in eMCAO rats treated with 2-BFI, rt-PA or 2-BFI + rt-PA. Quantitative analysis of **(C)** ZO-1 protein expression (n = 6) and **(D)** Occludin protein expression (n = 6). Quantitation of **(E)** MMP9 activation (n = 4) and **(F)** MMP2 activation (n = 4). Results are expressed as mean ± SEM. ^*^p < 0.05, ^**^p < 0.01, ^***^p < 0.001.

To investigate how 2-BFI and rt-PA might work synergistically to mitigate neuronal damage, we measured expression of the tight junction proteins Occludin and ZO-1. Rats treated with saline or rt-PA (6 h) alone had dramatically lower Occludin and ZO-1 levels than sham rats (**Figures 3A, C, D**). In contrast, rats treated with 2-BFI alone or 2-BFI + rt-PA (6 or 8 h) had higher ZO-1 and Occludin expression than rats treated with saline or rt-PA alone, with the 2-BFI + rt-PA (6 h) group showing the highest increase. These results suggest that 2-BFI and rt-PA protect against neurological damage by upregulating expression of tight junction proteins.

### Treatment With 2-BFI and rt-PA Increases Activation of MMP9 and MMP2 and Rescues Expression of Cerebrovascular Integrity Markers

We next examined the ability of 2-BFI and rt-PA to protect the ischemic area and rescue the penumbra area by measuring MMP9 and MMP2 activity in the ischemic penumbra. Ischemic rats treated with saline or rt-PA alone had higher MMP9 and MMP2 activation than sham animals, showing basement membrane disintegration and BBB integrity destruction. Treatment with 2-BFI + rt-PA (6 h) reduced MMP9 and MMP2 activation compared to rats treated with rt-PA alone, while treatment with 2-BFI + rt-PA (8 h) reduced only MMP9 activation (**Figures 3B, E, F**).

**Figure 4:**
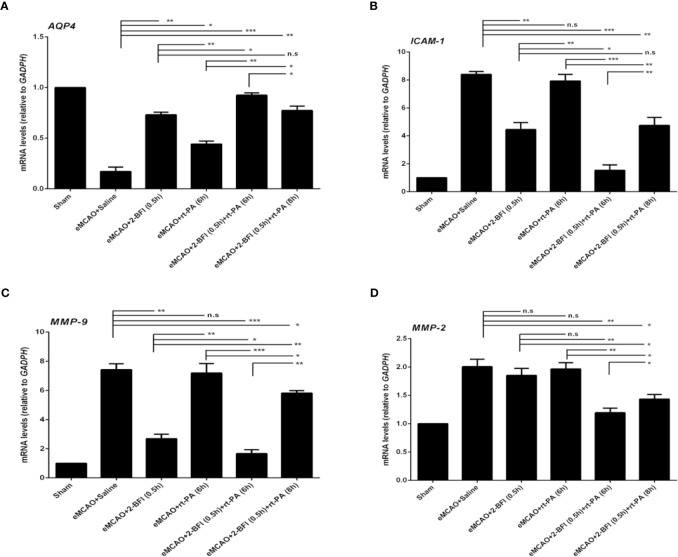
2-BFI and rt-PA rescue expression of BBB-associated genes in the penumbra. Expression of **(A)** *AQP4*, **(B)** *ICAM-1*, **(C)** *MMP-9*, and **(D)** *MMP-2* in eMCAO rats treated with 2-BFI, rt-PA or 2-BFI + rt-PA as assessed by qRT-PCR (n = 3-6 rats per group). Results are expressed as mean ± SEM. ^*^p < 0.05, ^**^p < 0.01, ^***^p < 0.001; n.s., not significant.

**Figure 5:**
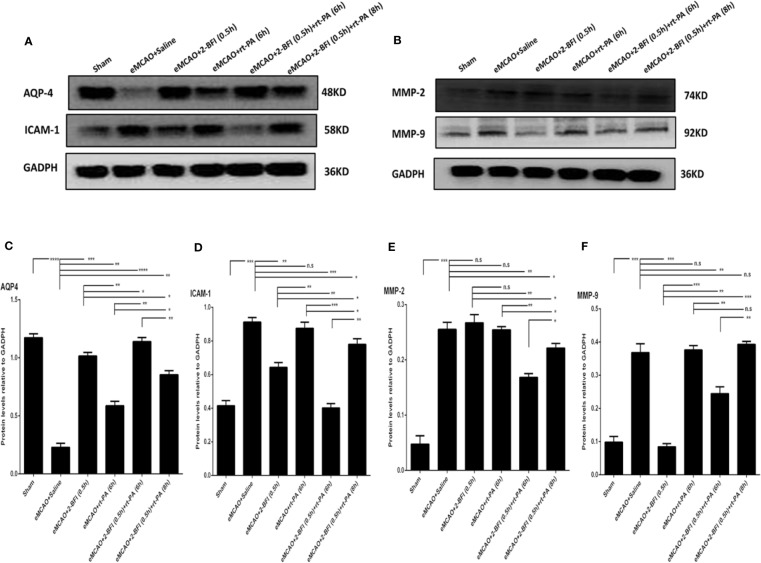
Combining 2-BFI and rt-PA restores levels of key proteins in the penumbra. Western blot showing expression of **(A)** AQP4 and ICAM-1 and **(B)** MMP2 and MMP9 in eMCAO rats treated with 2-BFI, rt-PA or 2-BFI + rt-PA. Quantitation of **(C)** AQP4, **(D)** ICAM-1, **(E)** MMP-2, and **(F)** MMP-9 protein levels. Results are expressed as mean ± SEM (n = 6). ^*^p < 0.05, ^**^p < 0.01, ^***^p < 0.001, ^****^p < 0.0001; n.s., not significant.

We next measured the expression of *AQP4*, *ICAM-1*, *MMP2*, and *MMP9*, which are markers of cerebrovascular integrity. Sham rats had the highest expression of *AQP4*, while saline-treated eMCAO rats had the lowest levels (**Figure 4A**). Rats treated with rt-PA alone had a slight increase in *AQP4* expression compared to saline-treated controls, whereas rats treated with 2-BFI + rt-PA (6 h) had the largest increase. Conversely, sham rats had the lowest expression of *ICAM-1, MMP2* and *MMP9*, while saline-treated animals had the highest (**Figures 4B–D**). Rats treated with 2-BFI with or without rt-PA (6 or 8 h) had lower *ICAM-1* expression than saline-treated controls, with the 2-BFI + rt-PA (6 h) group showing the largest decrease (**Figure 4B**). In contrast, rt-PA (6 h) alone did not reduce *ICAM-1* expression. Treatment with 2-BFI + rt-PA (6 or 8 h) down-regulated *MMP9* expression, with the 2-BFI + rt-PA (6 h) group showing the largest decrease. There was no difference in *MMP9* expression between rats treated with rt-PA (6 h) alone and saline-treated controls (**Figure 4C**). Rats treated with 2-BFI with or without rt-PA (6 or 8 h) had lower *MMP2* expression than saline-treated controls, but there was no difference in *MMP2* expression between saline-treated controls and rats treated with either 2-BFI or rt-PA (6 h) alone (**Figure 4D**). These gene expression changes were mirrored in the levels of the encoded proteins (**Figure 5**). Taken together, these findings show that 2-BFI and rt-PA act synergistically to protect cerebrovascular integrity.

## Discussion

The prevalence of ischemic stroke is rapidly increasing in developing countries but the only approved therapy is rt-PA (Han et al., 2012), which has a very narrow therapeutic window. Here, we show that combining rt-PA with 2-BFI can reduce the infarct volume, improve neurological function, reduce the number of denatured cells, decrease brain edema and maintain BBB integrity even when rt-PA is administered 6 h after ischemia onset. Molecular analysis suggests that 2-BFI exerts these effects by reducing cerebrovascular damage. Combining rt-PA with 2-BFI also reduced mortality and improved recovery after ischemic stroke. Combined treatment with rt-PA and 2-BFI could therefore substantially extend the 4.5-h window in current clinical practice (Hacke et al., 2008). It is important to mention that this combination therapy was unable to rescue BBB integrity in our model when rt-PA was given 8 h after onset of ischemia. Future experiments should be performed to determine the maximum time after stroke that treatment with 2-BFI and rt-PA is effective.

Animal and clinical studies show that while both early and delayed rt-PA are beneficial in treating stroke, rt-PA alone can also cause damage when administered more than 4.5 h after stroke onset (Kaur et al., 2004). Endogenous rt-PA is synthesized and released by cells within the vascular system. It exist in the vascular space, where it helps break down intravascular blood thrombi and fibrin deposits by converting plasminogen to plasmin (Liberatore et al., 2003). Emerging data suggests that under some conditions, both rt-PA and plasmin, which are broad spectrum protease enzymes, are potentially neurotoxic if they reach the extracellular space. With prolonged ischemia, the BBB is destroyed and there is a risk that rt-PA will enter the brain parenchyma and injure the neurovascular unit. The rt-PA is a ligand of the N-terminal domain (NTD) of the obligatory GluN1 subunit of NMDAR acting as a modulator of increasing extrasynaptic NMDAR surface dynamics and subsequent signaling (Lesept et al., 2016). This is demonstrated by a selected antibody named glunomab, leading to a selective reduction of the rt-PA-mediated surface dynamics of extrasynaptic NMDARs (Macrez et al., 2016). Thus rt-PA treatment amplifies the NMDA induced increase in intracellular calcium concentration and potentially could injure endothelial cells and provoke cell death (Nicole et al., 2001). At the same time, while L-arginine as a partial carrier of rt-PA, is a substrate for all isoforms of Nitric Oxide Synthase (NOS). It can increase the production of Nitric Oxide (NO), thus activate NMDAR and produce neurotoxicity (Huang et al., 1994). This suggests that the neurotoxic actions of rt-PA may exacerbate ischemic damage due to activation the NMDAR (Kaur et al., 2004; Rodier et al., 2014). Delayed thrombolysis exacerbates stroke-induced brain injury by triggering complex changes in gene expression and increasing MMP activation and basement membrane disintegration, leading to decreased BBB integrity and damage the central nervous system (Friedlander, 2003). This in turn leads to brain edema, increased vascular permeability and hemorrhage. And rt-PA also mediates neuronal destruction through plasmin proteolysis (Tsirka et al., 1995; Tsirka et al., 1996). Delayed rt-PA can also cause secondary thrombus formation in downstream microvessels (Jin et al., 2018). Consistent with this literature, we found that 2-BFI + rt-PA (8 h) was unable to rescue BBB integrity in our study, as the negative effects of rt-PA treatment at 8 h blocked the majority of the beneficial effects of 2-BFI. Therefore, it is important to identify neuroprotectants that can block excitotoxicity and protect BBB integrity, thereby reducing mortality and improving functional recovery after ischemic stroke. The neuroprotectants taurine, atorvastatin, baicalin and the 12/15-LOX inhibitor LOXBlock-1 have all shown promise as adjuvant therapies that can be combined with rt-PA (Zhang et al., 2009; Chen et al., 2017; Jin et al., 2018). An effective postischemic neuroprotective agent would permit the benefits of rt-PA and block all the negative effects, including reduced rt-PA neurotoxicity, reduced risk of hemorrhage, decreased reperfusion injury, amplified neuroprotective effect and increased therapeutic time window. These combined strategies are called cocktail treatments.

NMDARs are the most concerned neuroprotectant in recent years. Our current study extends our previous work showing that 2-BFI protects against brain injury by rapidly and reversibly blocking NMDAR-mediated calcium influx into neurons (Han et al., 2009; Han et al., 2010). 2-BFI acts similarly to memantine, another NMDAR antagonist which is approved for clinical use(Han et al., 2013). Activation of the NMDA receptor increases expression of MMP9 and MMP2, which are critical in the pathogenesis of post-ischemic BBB disruption and may be responsible for the disruption of tight junctions. Previous studies show that NMDAR receptor activation increases expression of MMP9 and MMP2 in a time-dependent manner, and that NMDAR antagonists reduce MMP9 and MMP2 expression (Neuhaus et al., 2011; Chen J.T. et al., 2016). Traditional noncompetitive antagonists such as katamine and MK801 are known to have neuroprotective effects. However, they also trigger adverse effects such as neuronal apoptosis, or they can aggravate brain injury (Vyklicky et al., 2014). In contrast, noncompetitive antagonists such as memantine and 2-BFI help maintain stability of the BBB and neurovascular unit by reducing the excitotoxic effects of excessive calcium influx (Chen Z. Z. et al., 2016; Wang et al., 2017). 2-BFI also has minimal side effects on physiological function because of the reversibility of the calcium influx blockade and its relatively fast off-rate from NMDARs. We previously reported that the protective effect of 2-BFI is stronger when it is administered early: 2-BFI treatment protected the brain in MCAO rats when it was administered within 5 h, but not 9 h, of cerebral ischemia/reperfusion (Zhang et al., 2018a). Previous studies have focused only on the neuroprotective effects of 2-BFI administered immediately after ischemia onset, which is rarely feasible in clinical settings. While delayed 2-BFI treatment can still provide neuroprotection up to 5 h after reperfusion. Besides, the drug could be injected to patients in the ambulance whether they have cerebral infarction or cerebral hemorrhage. 2-BFI is therefore a promising candidate for protecting against neuronal damage when treatment is delayed.

Combined treatment with 2-BFI extended the effective therapeutic window of rt-PA up to 6 h, with marked improvements in neurological function and reduced neuronal damage. This suggests that 2-BFI can block the negative effects of delayed rt-PA treatment by maintaining BBB integrity and stability. The BBB consists of endothelial cells, astrocytic, pericytes, tight junction proteins and the basement membrane, and acts as a specialized cerebral barrier. It is critical for maintaining the correct microenvironment for proper neuronal function (Zlokovic et al., 2005), with destruction of the BBB following ischemic brain injury leading to hemorrhage and brain edema. The neuroprotective effects of 2-BFI may be mediated by down-regulation of ICAM-1, MMP2, and MMP9 and up-regulation of tight junction proteins. MMP activation leads to degradation of the extracellular matrix around the neurovascular unit, exposing the BBB microcirculation and increasing risk of brain edema and hemorrhage (Rosenberg and Yang, 2007; Hjort et al., 2008). Activation of MMP2 and MMP9 degrades the basal lamina and tight junction proteins involved in cell-cell adhesion, thereby compromising BBB integrity (Liu et al., 2011; Chen J.T. et al., 2016). Some studies have reported a positive correlation between BBB permeability and expression of tight junction proteins, specifically Occludin and ZO-1 (Srivastava et al., 2013). Loss of tight junction proteins on the cytomembrane were also observed in the damaged BBB after cerebral ischemia (Fernandez-Lopez et al., 2012). ICAM-1 mediates intercellular adhesion, promotes leukocyte aggregation, and increases pro-inflammatory cytokine expression, all of which are known to mediate thrombosis formation. Delayed rt-PA treatment increases the risk of secondary thrombosis (Fitzgerald et al., 1988; Liu et al., 2006), which can increase the infarction area. AQP4 is the major water channel in brain perivascular astrocyte processes and transports water bidirectionally (Han et al., 2015). It plays a pivotal role in cerebral edema formation and BBB integrity (Abbott et al., 2006). Studies have reported both increases and decreases in AQP4 expression after vasogenic brain edema (Papadopoulos et al., 2004; Cheng et al., 2018; Ozen et al., 2018). We found that ischemia downregulated AQP4, while treatment with 2-BFI and rt-PA up-regulated AQP4 in the ischemic penumbra area. This is consistent with our previous findings showing decreased AQP4 during the early stages of edema formation in the peri-infarct area(Zhang et al., 2018b). The above changes in gene and protein expression may explain how 2-BFI stabilizes the BBB to alleviate brain injury after stroke.

Treatment with rt-PA may have neurotoxic effects in the brain, including direct vasoactivity, amplification of intracellular Ca^2+^ conductance, and activation of extracellular MMP proteases (Oka et al., 2009). Recent evidence suggests that rt-PA is potentially neurotoxic if it reaches the extracellular space (Kaur et al., 2004). Some experiments also suggest that rt-PA may cause neuronal injury through extravascular protease activity in ischemia. These effects may increase excitotoxicity, further damage the BBB, and worsen edema and cerebral hemorrhage (Nagai et al., 1999). Treatment with rt-PA further amplifies the NMDA-induced increase in intracellular calcium and could potentially lead to cell death.

Our results provide strong evidence that 2-BFI can counteract several of the negative effects of delayed rt-PA treatment, improve overall therapeutic efficacy and protect BBB integrity, thereby prolonging the therapeutic window. Monotherapy with rt-PA (6 h) down-regulated Occludin and ZO-1; up-regulated ICAM-1, MMP2, and MMP9; and inhibited MMP9 and MMP2 activation. These effects were reversed when 2-BFI was administered in combination with rt-PA (6 h) and correlated with reduced brain edema and Evans blue extravasation. Treatment with 2-BFI appears to inhibit side effects of rt-PA, perhaps by preventing Ca^2+^ influx and antagonizing glutamate-mediated neurotoxicity. This would translate to a smaller ischemic lesion volume even if rt-PA were not administered until 6 h after stroke, presumably because 2-BFI would protect BBB integrity.

Our study has several limitations. The therapeutic window for stroke in rodents can not be compared with the therapeutic window in humans. Healthy adult rats were used in this study, whereas most stroke patients tend to be older. We did not explore the long-term effects of 2-BFI treatment, which is currently being investigated by our group. Future studies should examine the therapeutic window of this combination therapy in older animals and humans. On the other hand, mechanisms to explain the effects observed in the present study are not yet elucidated. We should seek to clarify how 2-BFI confers neuroprotection. Despite these limitations, this is a study providing evidence that 2-BFI can alleviate brain injury and BBB damage caused by delayed thrombolysis.

## Conclusions

In summary, our results suggest that combining 2-BFI with rt-PA can protect against brain damage by reducing ischemic damage and BBB permeability, even when rt-PA treatment is delayed until 6 h after stroke. Combining 2-BFI and rt-PA is a promising therapy that may extend the therapeutic window for stroke victims. Future studies should examine the therapeutic window of this combination therapy and seek to clarify how 2-BFI confers neuroprotection.

## Data Availability Statement

The datasets generated for this study are available on request to the corresponding author.

## Ethics Statement

The animal study was reviewed and approved by the Institutional Animal Care and Use Committee of Wenzhou Medical University.

## Author Contributions

All authors had full access to all the data in the study and take responsibility for the integrity of the data and accuracy of the data analysis. The roles of the authors are as follows: LZ: writing the original draft and methods. SX, XW, and JiaoC: experimental studies. XG and ZZ: manuscript writing, reviewing, and editing. YC and JY and JianC: supervision; ZH: funding acquisition.

## Funding

Work was supported by the National Natural Science Foundation of China (81571114, 81771267, 81701426), the Natural Science Foundation of Zhejiang province (LY17H090014), the Medical and Health Research Science and Technology Plan Project of Zhejiang Province (2018KY523), and the Public Welfare Science and Technology Plan Project of Wenzhou City (Y20140686, Y20170151 and Y20180132).

## Conflict of Interest

The authors declare that the research was conducted in the absence of any commercial or financial relationships that could be construed as a potential conflict of interest.
